# Sexual Dysfunctions among Veterans with and without PTSD

**DOI:** 10.3390/healthcare11131861

**Published:** 2023-06-27

**Authors:** Marina Protuđer, Aleksandra Stevanović, Marina Letica-Crepulja

**Affiliations:** 1 Department of Psychiatry and Psychological Medicine, Faculty of Medicine, University of Rijeka, 51000 Rijeka, Croatia; marina2ri@yahoo.com (M.P.); aleksandras@medri.uniri.hr (A.S.); 2 Department of Psychiatry, Clinical Hospital Center Rijeka, Referral Center for PTSD of the Ministry of Health of the Republic of Croatia, 51000 Rijeka, Croatia; 3 Department of Basic Medical Sciences, Faculty of Health Studies, University of Rijeka, 51000 Rijeka, Croatia

**Keywords:** posttraumatic stress disorder, sexual dysfunction, veterans

## Abstract

Background: Research on the relationship between posttraumatic stress disorder (PTSD) and sexual dysfunctions (SD) has clearly recognized the association among these conditions. The main objective of this research was to compare the levels of the overall and the level of certain domains of sexual functioning among veterans with and those without PTSD. Methods: Two hundred and fifty veterans with PTSD and 187 veterans without PTSD were included in the comparative study. The following assessments were conducted: LEC-5, PCL-5, ITQ, IIEF, PEDT, and MINI. Results: Veterans with PTSD had significantly higher levels of all types of SD in the PTSD group compared with the non-PTSD veterans. Veterans with PTSD more frequently experienced psychiatric and somatic comorbidities and use of medication that may contribute to the occurrence and severity of SD. Conclusions: The present study emphasized that SDs are an important issue among patients with PTSD. The study comprehensively accounted for conditions that may contribute to the occurrence and severity of SD among veterans with PTSD. Future directions of the research that could further improve the healthcare of the patients were indicated.

## 1. Introduction

The rates of sexual dysfunctions (SDs) among patients with posttraumatic stress disorder (PTSD) are much higher than in the general population, varying from 8% to 89%, respectively. The interesting finding made so far has been that studies reporting the low prevalence were mainly based on the numbers of diagnoses observed from medical files, whereas those showing the high prevalence were based on direct interviews and questionnaires [1]. What can we learn from that? Unfortunately, the finding indicates that the huge problems of the comorbidities underlying PTSD and SD is neglected on all levels of healthcare, leaving patients unrecognized with their personal suffering, possible worsening of condition, and without adequate treatment. We can expect further worsening of the problem as the number of persons suffering from PTSD is likely to increase in the years to come due to the rising numbers and intensity of interpersonal conflicts, wars, natural and man-made disasters, and other potentially traumatic circumstances worldwide. Consequently, it is of the utmost importance to improve the awareness of the general practitioners and other healthcare professionals who are not specialists in mental health on the sexual problems of those who suffer from PTSD.

SD among veterans with PTSD have been almost totally neglected in research until an increase of interest occurred in recent years. Veterans have been regularly exposed to multiple traumatic experiences, but studies have shown that SDs are much more prevalent among veterans with PTSD than in veterans without PTSD, or in the general population [1,2,3,4,5,6,7,8,9,10,11,12,13]. These data imply that PTSD, rather than traumatic experiences determine the type and severity of sexual problems [1,10,14]. In other words, we can say that all veterans experience potentially traumatic events during their war deployment, but problems in sexual functioning will occur more frequently among those who experience negative mental health consequences, such as PTSD. On the other hand, several prior studies have identified the direct impact of trauma on sexual problems among male veterans irrespective of their PTSD symptoms [15,16]. The recent study of Kolaja and colleagues found that PTSD symptoms mediated the impact of traumatic experiences on sexual health problems among veterans [17]. 

What kind of SDs are related to PTSD? Studies have so far revealed the association of PTSD with overall sexual function, as well as impairments in the specific domains of sexuality, such as activity, desire, arousal, orgasm, and satisfaction with sexual life [10,18]. The recent comprehensive review on SD among veterans and military personnel confirmed that PTSD is clearly associated with impairments in overall sexual functioning, particularly with sexual desire, sexual satisfaction, and sexual distress, with mixed results when examining relationships between PTSD and sexual arousal, erectile dysfunction, premature ejaculation, delayed ejaculation, and frequency of sexual activity [19]. Limitations of the existing research has been attributed to neglecting the comorbidities and use of medications that are well-known contributing factors to impairments in sexual functioning [19]. 

Comorbidities with psychiatric and somatic conditions, and the use of a variety of medications are very common among veterans with PTSD. Some of those conditions carry a significant burden for the worsening of sexual functioning [20,21]. Regarding physical health, SDs are associated with common health conditions such as diabetes mellitus [22,23] and hypertension [24]. SDs are highly prevalent among patients with psychiatric disorders [25,26], and may be caused by not only the psychopathology, but also by the pharmacotherapy used in treatment [27,28,29]. Nearly all psychoactive substances influence sexual functioning. Treatment-emergent sexual dysfunction adversely affects the quality of life and may reduce treatment adherence [27]. In addition to psychotropic drugs, several classes of prescription drugs contribute to SDs in the general population, and antihypertensives are most commonly used among them [30].

Almost 30 years after the Homeland War in Croatia (1991–1995), veterans still suffer from numerous health problems. In recent periods, case reports [31] and research articles [5,6,18] on SDs among Croatian veterans with PTSD have been published revealing a huge proportion of undiagnosed and untreated SDs. The main objective of this research was to assess whether higher levels of SD are related to PTSD or not through comparing the group of veterans with and the veterans without PTSD. Another objective of our research was also to comprehensively explore the relationship between all types of SD, comorbidity, the use of medication, and the presence of PTSD. 

## 2. Participants and Methods

### 2.1. Participants and Procedure

A total of 437 war veterans participated in this study. The group of veterans with PTSD (N = 250) was recruited from a pool of patients referred from the Referral Center for PTSD of the Ministry for Health of the Republic of Croatia within the Clinical Hospital Center (CHC) Rijeka. The control group consisted of comparable male combat veterans without PTSD (N = 187). Target sampling [32] was used to ensure an adequate representation of the control group, and sampling bias was minimized as much as possible using linkage sampling [33] and snowballing. The inclusion criteria for the study were participation in military activities during the Homeland War and having experienced at least 1 combat-related traumatic event, meeting the diagnostic criteria for war-related PTSD as defined in the Diagnostic and Statistical Manual of Mental Disorders, Fifth Edition (DSM-5) [34], male gender, and being of an age below 65. Thirteen of those whom we approached refused to participate, two patients did not complete the questionnaires, and three patients did not meet the diagnostic criteria for PTSD and were thus not included in the study.

Exclusion criteria were active psychosis, and moderate or high suicide risk, measured using the Mini-International Neuropsychiatric Interview (MINI) for DSM-IV [35], and deformities of the genital organs. 

Assessments included clinical interviews and self-report questionnaires. Three psychiatrists and 2 psychologists performed the interviews with the participants. A sociodemographic questionnaire was created for the study purposes. The assessment was usually completed in one session, with an average of 2 h. The study was approved by the Ethics Committees of the Faculty of Medicine, University of Rijeka. All participants were informed of the purposes and procedures to be undertaken in the study and were given detailed information on the risks and benefits. Written informed consent was obtained from all participants included in the study. 

### 2.2. Measures

#### 2.2.1. Assessment of PTSD and War Trauma

PTSD symptoms were assessed with the PTSD Checklist (PCL-5) and Criterion A [36]. PCL-5 is a self-report checklist with 20 items reflecting PTSD symptoms according to the DSM-5 criteria for PTSD. Participants rated each item on a scale from 1 to 5 to indicate the degree of disturbance caused by that symptom during the past month. Overall symptom severity score was defined as the total sum (range 0–80). To establish the presence of PTSD, we used the overall score of 33 or higher, which is in line with previous studies [37,38]. PCL-5 is reported to display good psychometric properties [37,38]. The Cronbach alpha in our study for total PCL-5 was 0.97. The Criterion A measure was included in the assessment according to the criteria of DSM-5 [34].

To assess the presence of war-related traumatic events we used the Life Events Checklist for DSM-5 (LEC-5) [39], a self-reported measure assessing 17 possible traumatic events that occurred throughout a participant’s lifetime. The exposure was rated as follows: happened to me; witnessed it; learned about it; part of my job; not sure; and does not apply, respectively. The item of interest in this study was the exposure to combat or exposure to a warzone (in the military or as a civilian) on the personal level (happened to me), which was found to be positive for all included veterans. The LEC demonstrated adequate psychometric properties as a stand-alone assessment of traumatic exposure [40].

#### 2.2.2. Assessment of Sexual Function 

Two measures were used to assess sexual function. The International Index of Erectile Function (IIEF) [41] was used to assess five domains of male sexual functions: erectile function (6 items), intercourse satisfaction (3 items), orgasmic function (2 items), sexual desire (2 items), and overall satisfaction (2 items). Participants rated the level of sexual functioning in the last four weeks on a 5-point Likert scale. The score for erectile dysfunction can be classified as severe, moderate, and mild or no erectile dysfunction. The IIEF has good psychometric properties [41,42,43], and the internal validity in our study was satisfactory with Cronbach alphas from 0.89 to 0.96 for the specific domain, respectively. 

To assess the presence and severity of PE, we used premature ejaculation diagnostic tool (PEDT) [44,45]. PEDT is a 5-item self-report questionnaire, with each item rated on a 5-point Likert scale. The score is the sum of all the answers (range 0–20), with higher scores suggesting more difficulties with premature ejaculation. The PEDT has satisfactory psychometric properties [44,45], and the Cronbach alpha in our study was 0.87.

#### 2.2.3. Assessment of Comorbidity and Medication Use 

The Croatian version of the Mini-International Neuropsychiatric Interview (MINI) for DSM-IV [35], was used to assess 17 of the most common psychiatric disorders observed in DSM-IV. Comorbid somatic diseases and their related health problems were assessed through self-reporting and were later classified in accordance with the International statistical classification of diseases and related health problems—10th revision, Fifth edition (ICD-10) [46]. Reported medication in current use was classified according to the Anatomical Therapeutic Chemical (ATC) classification [47]. 

### 2.3. Data Analysis

Data was analyzed with Statistica software, version 12 (Dell Inc. Inc., Tulsa, OK, USA) and were presented as N (%), M (sd), or median as appropriate. Results on the dependent variables (sexual dysfunctions and PTSD symptom intensity) did not meet assumption of normal distribution as assessed using the Kolmogorov–Smirnov test and boxplots. To examine between group differences, we used the Mann–Whitney U test, chi-square test, and Fisher’s exact test for variables with small cell sizes. To calculate the extent of correlation between the variables of interest we used the Spearman coefficient. Probability significance was set as *p* ≤ 0.05.

## 3. Results

### 3.1. Sociodemographic Data

The average age of war veterans included in the study was 53.7 (SD = 6.27) years, with the range from 44 to 65 years. respectively. Most of them were married (66.6%), with secondary education (77.2%), and perceived their economic status as medium (70.6%). Veterans without PTSD were 3.7 years older on average (PTSD: 52.1 years, non-PTSD: 55.8 years, respectively; t = 6.415, *p* < 0.001), more likely to be in a relationship (PTSD n = 218 (87.2%), non-PTSD n = 175 (93.6%)); Fisher’s exact test *p* < 0.001), to have a medium economic status (PTSD n = 158 (63.3%), non-PTSD n = 150 (80.2%), χ2 = 14.678, *p* < 0.001), and to have a higher level of education (PTSD n = 20 (8.1%), non-PTSD n = 41 (21.9%); χ2 = 16.846, *p* < 0.001) compared to veterans with PTSD.

#### 3.1.1. Trauma Exposure and PTSD

The average intensity for the overall PTSD symptoms among war veterans was 38.7 (sd = 24) within the range from 0 to 80, respectively. As expected, veterans with PTSD had a significantly higher intensity of the overall PTSD symptoms (mean = 57.4, sd = 10.68) compared to veterans without a provisional diagnosis of PTSD (mean = 13.7, sd = 9.99). Both groups met the criteria for at least one war-related traumatic event and have had high exposure as assessed by Criterion A. However, the PTSD group was deployed significantly longer (U = 16,321, *p* < 0.001) compared to the non-PTSD group, with a median of 26.5 months compared to the median of 18 months in the period from 1919 to 1996, respectively. The average duration of active participation in the Homeland War was 27.1 (18.98) months, ranging from 1 month to 70 months, respectively. 

#### 3.1.2. Sexual Dysfunction 

The results suggest a mild to moderate erectile dysfunction (mean = 18.8, sd = 9.74) in the overall sample (n = 433). The average score for orgasmic function was 6.7 (sd = 3.25), (theoretical maximum = 10), sexual desire 5.6 (sd = 2.47) (theoretical maximum = 10), intercourse satisfaction 7.6 (sd = 4.55) (theoretical maximum = 15), and for overall satisfaction 7 (sd =2.32) (theoretical maximum = 10), respectively. Although there is no clear classification of the level of dysfunction for the domains on the IIEF other than erectile dysfunction, the results are comparable to those provided by the authors of the IIEF. Rosen et al. [41] reported similar averages in their validation study among the sample of men diagnosed with sexual dysfunction. It is therefore possible to conclude that the sample of war veterans in our study has a significant level of SD, comparable to those of men with a SD diagnosis. The average score on the PEDT was 6.2 (4.86) within the range from 0 to 20, respectively, suggesting a significant level of premature ejaculation.

### 3.2. Sexual Dysfunction in Relation to PTSD

To assess for the association between PTSD and SD, we compared two groups in different domains of sexual functioning (Table 1). Veterans with PTSD had significantly lower scores on erectile dysfunction, orgasmic function, sexual desire, intercourse satisfaction, and overall satisfaction compared to the non-PTSD group, thereby suggesting a higher level of SD in the PTSD group. Non-PTSD veterans had significantly lower scores on the PEDT, indicating less problems with premature ejaculation compared to the PTSD group. 

**Figure 1:**
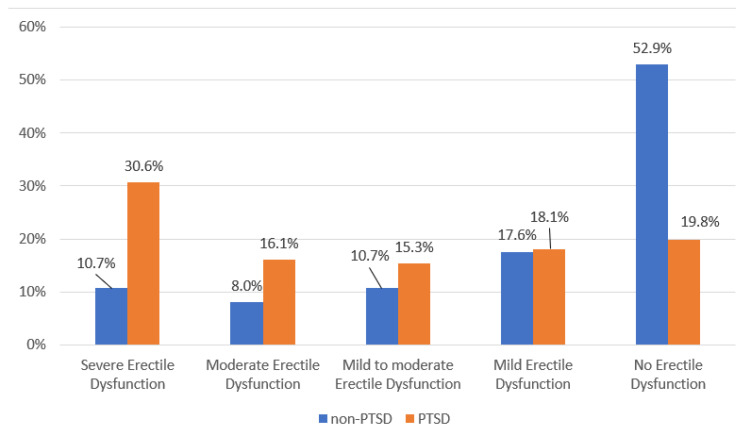
Participation distribution according to the level of erectile dysfunction.

Scores for erectile function on the IIEF can be categorized depending on the level of dysfunction. Veterans with PTSD were more likely to have severe and moderate erectile dysfunction compared to the non-PTSD veterans (χ = 61, *p* < 0.001). More than half of the veterans without PTSD experienced no erectile dysfunction at the time of measurement compared to 19.8% veterans with PTSD (Figure 1).

Even though the rates of SDs were found to be significant in the overall sample, comparisons between the PTSD and non-PTSD groups revealed the presence of the significant association between PTSD and SD.

### 3.3. Comorbidity and Drug Use 

All the participants from the PTSD group had sought psychiatric help at some point, with 22% of them having done so in the period from 1991–1995, while the war was ongoing. In the non-PTSD group, 17 (9.9%) veterans had sought psychiatric help, with 10 of them having done so during the war.

Participants without PTSD did not meet the criteria for any psychiatric comorbid disorders measured using the MINI. Among participants with PTSD, the most common comorbid psychiatric disorders were current (n = 50), lifetime major depressive episode (MDE; n = 82) (overall 52.8%), and panic disorders (current and lifetime 29.2%). Current suicidality (low suicide risk) was present in 6 (2.4%) veterans. At least one of the other anxiety disorders was present in 45 (18%) participants (agoraphobia: ten, social phobia: five, obsessive-compulsive disorder: four, and generalized anxiety disorder: twenty-six, respectively). Nine participants (3.6%) reported alcohol use disorder and six (2.4%) had drug-related disorders in the last year. Three participants were classified with antisocial personality disorder, and one with an eating disorder. None of the participants were classified with a current psychotic disorder although two have lifetime psychotic disorder. 

The most common self-reported non-psychiatric diseases in the overall sample were diseases of the circulatory system (n = 169, 38.7%), followed by endocrine, nutritional, and metabolic diseases (n = 81, 18.5%), and diseases of the musculoskeletal system and connective tissue (n = 55, 12.6%). When it came to the diseases that have already been recognized as significantly related to SD, the most commonly self-reported in our sample was essential hypertension, which affected 153 (35%) participants, followed by diabetes mellitus (n = 51, 11.7%), diseases of lipoprotein metabolism (n = 34, 7.8%), and hyperplasia of the prostate (n = 10, 2.3%). Some of the conditions known to influence SD were rarely present in our sample: sleep apnea (four), malignant neoplasm of testis (two), epilepsy (two), other hypothyroidism (four), multiple sclerosis (one), Parkinson’s disease (one), and autoimmune thyroiditis (one), respectively. Veterans with PTSD were significantly more likely to have had essential hypertension (n = 107 (42.8%) vs. n = 46 (24.6%), respectively; χ2 = 15.576, *p* < 0.001) and diabetes (n = 38 (15.2%) vs. n = 13 (7%), respectively; χ2 = 7.06, *p* = 0.008) compared to the non-PTSD group. There was no significant difference observed in the disorders of lipoprotein metabolism (n = 20 (8%) vs. n = 14 (7.5%), respectively; χ2 = 0.843, *p* = 0.843) or hyperplasia of the prostate (n = 6 (2.4%) vs. n = 4 (2.1%), respectively; Fisher’s exact test *p* = 1) between the groups. Significance of differences were confirmed using the Fisher’s exact test.

In the PTSD group, 94.8% of participants used at least one psychotropic drug, compared to only 14 (17.5%) of non-PTSD participants. Among those who were taking psychotropic medication, 36 (14.6%) had a single drug, 76 (30.9%) had a combination of two psychotropics, 75 (30.5%) had a combination of three psychotropics, and 59 (24%) had a combination of four or five psychotropics, respectively. The average number of psychotropic drugs in the PTSD group was 2.59 (median = 3), and in the non-PTSD group 0.1 (median = 0).

The most common psychotropics in the overall sample were anxiolytics, with 202 (46.2%) participants taking at least one anxiolytic therapy. Furthermore, 133 (30.4%) participants used at least one antidepressant, 73 (16.7%) of them used at least one hypnotic and sedative, 90 (38.5%) used at least one antiepileptic, and 63 (14.4%) at least one antipsychotic, respectively. Only three participants had used drugs in addictive disorders (such as methadone). There was a large discrepancy observed between psychotropic medication between the groups (Table 2).

The most common self-reported non-psychotropic medication was related to cardiovascular therapy (31%), followed by medications for the musculoskeletal system (12.8%). When it came to drugs that were previously recognized as related with SDs, the most common was antihypertensive therapy, with 116 (26.5%) participants using at least one antihypertensive. At least one lipid-modifying agent therapy was used by forty-six (10.5%) participants, drugs used in diabetes by twenty-six participants (5.9%), H2 blockers by six, antihistamines by five, corticosteroids for systemic use and immunosuppressants by two, and drugs for erectile dysfunction by only one participant. Differences in non-psychotropic drugs with a frequency of at least five are presented in Table 2.

### 3.4. Correlates of Sexual Dysfunctions

Correlation coefficients between SDs and variables presenting sociodemographic data, comorbidity, drugs used, PTSD symptom severity, and war deployment duration are presented in Table 3. 

## 4. Discussion

The most important finding of this research was that veterans with combat-related PTSD experienced significantly more frequent sexual difficulties than the veterans without PTSD in all varieties of impairments in the specific domains of sexuality (desire—arousal—orgasm—resolution), as well as in the overall personal satisfaction and satisfaction with intercourse. As the DSM-5 [34] provides classifications for four male SDs, including male hypoactive sexual desire, erectile dysfunction, delayed ejaculation, and premature ejaculation, these results imply that combat-related PTSD increases the risk for the development of all the SDs listed in the actual international classifications of diseases, including premature ejaculation, which has been less examined compared to that of other SDs in previous research. The practical value of this finding was the possibility to narrow the focus on a particular group of population exposed to trauma, i.e., veterans with PTSD where we can expect more problems in sexual functioning. The most common problem for the healthcare professionals when our society is hit with a crisis is not knowing where to start and what to expect. The finding may contribute to a better triage of the patients for the interventions and treatment of SDs. These findings are consistent with the existing systematic reviews which confirmed a correlation between PTSD and SD [9,10,11]. Yehuda et al. in their review additionally proposed PTSD-related biological, cognitive, and affective processes that may result in impaired sexual functioning [10]. The most recent review on the relationship between PTSD and SD among veterans and military personnel confirmed that PTSD was associated with an increased risk of experiencing at least one sexual difficulty. Regarding the specific impairments, PTSD was most clearly associated with impairments in sexual desire, sexual satisfaction, and sexual distress, with mixed results when examining relationships between PTSD and sexual arousal, erectile dysfunction, delayed ejaculation, premature ejaculation, and frequency of sexual activity [19]. Accordingly, our results confirming the clear association of PTSD and all types of SD in the veteran population present a relevant contribution to the rising body of research that should pave the way for improvements in the diagnostics and treatment of patients with co-occurring PTSD and SD. Regarding the worldwide increment of the negative mental health impacts of psychological trauma, the fact that only one participant with PTSD had the diagnosis of SD and only that same participant was adequately treated is the reason for serious concern. The reasons for this could be numerous. We could assume that some healthcare professionals are not aware of the increased risk for sexual problems among veterans with PTSD, as the research had been sparse with confronting results until recently [19]. Some of them may have a problem with the lack of time for in-depth inquiries about the sexual functioning. Healthcare professionals may also avoid talking about sexuality because of personal reasons. For example, the reasons could be anxiety and shame while talking about sexuality or other intimate themes, insufficient skills for leading the interview about sexual functioning, strict religious beliefs or rules, social and cultural customs, opposite gender of the patient, and concerns about the patient reactions. From the patient’s side, the stigma of having a SD, shame, or discomfort may be the main reasons for not revealing, or even withholding or denying the problems in sexual functioning. In the veteran population, this can be related to the attitudes about masculinity that are closely tied with sexual functioning and the personal sense as a “real man” with the consequences of a low self-esteem and negative beliefs about themselves. 

Regarding the level of SD in both groups of participants, the scores on the IIEF permitted the classification of the severity for erectile dysfunction as severe, moderate, and mild or no dysfunction. The scores in our sample indicated that veterans with PTSD were significantly more likely to have severe and moderate erectile dysfunction compared to non-PTSD veterans, while the frequency of mildly occurring sexual difficulties was similar. Previous research showed consistent results in that participants with symptomatic PTSD exhibited significantly more severe symptoms related to erectile dysfunction [4,48].

The results revealed that participants from the PTSD group experienced extensive comorbidities with depression, anxiety disorders, and to a lesser extent, substance-related disorders. Unlike them, participants without PTSD did not meet the criteria for any psychiatric comorbidity disorders. It has been well-known that patients with PTSD commonly experience comorbid psychiatric conditions. The data from epidemiologic surveys have indicated that depression, anxiety disorders, and substance-related disorders are the most prevalent comorbid psychiatric disorders among PTSD patients, and in turn, all of them increase the risk for SD [26,31]. Kotler et al. have found high levels of comorbid panic disorder, depression, and anxiety in patients with PTSD, and they consider that could be the reason for more frequent sexual dysfunctions [3]. Consistent with the previous reports of a clear association between depression and sexual dysfunction [49,50], our findings demonstrated that depression was associated with all SDs except for premature ejaculation. The clinical features like anhedonia, the inability to experience positive emotions, fatigue, and poor self-esteem may contribute to the impairment of normal sexual functioning in patients with depression [51,52]. Anxiety plays an important role in the pathogenesis and maintenance of SDs. Co-occurring anxiety disorders with PTSD increase the risk for SD, as SDs are often found among patients with anxiety disorders (e.g., 75% among patients with panic disorder) [12]. The result that alcohol-related disorders were less frequent and not clearly associated with any of the examined SDs in our sample may be surprising on the first sight, as previous studies have indicated a high prevalence of SD in men with chronic alcohol use [53,54,55], and alcohol-related disorders appeared to be a significant predictor of SD [18]. This finding in our research could be related to the sample of treatment-seeking PTSD patients mostly included in the treatment for PTSD as a primary diagnosis. Veterans with alcohol dependence or severe problems with heavy drinking usually have difficulties in persisting to treatments focused primarily on PTSD, as they are preoccupied with craving, problems with abstinence, social deterioration etc. These patients are included in separate programs for comorbid disorders/dual diagnoses that cover both conditions simultaneously, but PTSD on the lower level of interventions are adjusted to the patient capacities. 

Several physical conditions influence sexual functioning. In our sample, essential hypertension and diabetes mellitus were the most prevalent in the overall sample and were significantly more frequent among veterans with PTSD than among those without a PTSD diagnosis. Hypertension appeared to be associated with all types of SD, and diabetes mellitus only with premature ejaculation. High blood pressure has already been confirmed as a risk factor of SD [24]. Possible explanations are the common pathophysiologic pathways, such as atherosclerosis and endothelial dysfunction, and the medicine used for hypertension is also known for its adverse effect sexual functions [56]. Additionally, vasculogenic erectile dysfunction is considered as part of a systemic vasculopathy, and has a known relation with cardiovascular risk factors, including hypertension, diabetes, dyslipidemia, and smoking [57]. The relationship between premature ejaculation and diabetes mellitus has also been previously reported. The levels of dysfunction are related to the duration of diabetes mellitus, severity, and stability of metabolic control [58]. On the other hand, a close relationship between erectile dysfunction and premature ejaculation exists with anxiety as a mediator [59]. Low trust in erectile function can lead to an elevated performance anxiety, causing rushed intercourse and premature ejaculation [18,59].

Almost all participants with PTSD used at least one psychotropic drug. In comparison with the non-PTSD group, those with PTSD used them five times more frequently. The most common psychotropics in the overall sample were anxiolytics followed by antidepressants. The overuse of anxiolytics in the same post-conflict setting has already been reported [60], as well as their association with SD [27]. Benzodiazepines have been associated with a decreased libido and an impaired arousal and orgasm, although the link seems to be dose-related [27]. In our study, the use of antidepressants was found to be correlated with all types of SD. This result should be interpreted in the light of the clinical guidelines which recommend antidepressants as the medication of the first line in the management of PTSD [61,62]. The results are consistent with meta-analysis that confirmed increased rates of SD among patients treated with antidepressants [29]. The impact of antidepressants on neurotransmitter pathways, such as the catecholamine (dopamine and norepinephrine), and indoleamine (serotonin) systems, may have a major role in explaining SDs among patients involved in treatment with antidepressants. For example, dopamine impacts sexual desire and motivates sexual behavior, norepinephrine enables sexual arousal and vasocongestion, and serotonin causes vasocongestion, shutting out arousal. There is evidence that serotonin may influence nitric oxide function decreasing genital sensation as a result [27].

The most common non-psychotropic medication was antihypertensive therapy, and it appeared to be related with all types of SD except for PE. La Torre et al., in their review found that most of the studies recognized antihypertensive drugs with a key role in sexual dysfunctions induced by cardiovascular drugs. There is also evidence suggesting that older antihypertensive drugs (diuretics, beta-blockers, and centrally acting agents) have a more negative impact on erectile function when compared to newer agents [30].

What could be implications of the findings on the quality of healthcare? It could be the raised awareness of the issues of the SD and PTSD comorbidities among the healthcare providers and users. Professionals may strive to overcome the personal reasons to question patients about their sexual functioning, as the research strongly confirms that patients with SD want their doctors to initiate talks about sexual health [63]. This research may contribute to a better triage in the aftermath of crisis, giving a better insight to professionals on what types of negative mental health consequences and comorbidities could prevail. The comprehensive approach widens the insight of very frequently contributing factors to impairments in sexual functioning in the PTSD population, such as comorbidities with psychiatric and somatic conditions and the use of a variety of medications. Knowledge about the existence of the problem, better recognition, and assessment may be the basis for the improvement of the treatment of SD among patients with PTSD.

### 4.1. Strengths

This research had strengths worth noting. The participants’ index trauma was assessed, expanding our understanding of the extent to which trauma type may moderate the relationship of PTSD with sexual functioning. Comparisons with the matching control group of veterans with the same traumatic experiences but without the satisfied required criteria for the diagnosis of PTSD allowed more firm conclusions to be made about the association between PTSD and SD in the veteran population. The study comprehensively accounted for the psychiatric and physical comorbidities, and the use of all varieties of medications that may contribute to the occurrence and severity of SD among the veterans with PTSD. The data related to SD were systematically collected as they covered a broader range of possible sexual health problems as well as perceived sexual satisfaction. 

### 4.2. Limitations

This study had several limitations. The study was limited to male veterans, and thus the findings cannot be generalized to the general population or the female population. Data were cross-sectional and did not allow insights in causation. The study relied on self-reports and was therefore subject to participant bias. Self-reports of sexual functioning may be under- or overreported due to stigmatization.

## 5. Conclusions

The present study showed that veterans with combat-related PTSD experienced significantly more frequent sexual difficulties than veterans without PTSD. The study comprehensively accounted for psychiatric and physical comorbidities, and the use of all varieties of medications that may contribute to the occurrence and severity of SD among veterans with PTSD. These results are a relevant contribution to the rising body of research that should pave the way for the improvement in the healthcare of veterans with co-occurring PTSD and SDs. 

Future direction of the research that could improve the healthcare of the patients should be focused on identifying the most important factors that prevent recognizing and diagnosing SD among patients with PTSD. Widening the research on other groups of traumatized people would further contribute to the generalizability of the results. On the level of primary healthcare, research could be focused on getting more qualitative data from patients regarding the context of trauma and the nuances of SDs. Longitudinal research could enable better insights to be obtained in the dynamics of development and the course of comorbidity of SDs and PTSD.

## Figures and Tables

**Table 1:** Sexual dysfunctions among veterans with PTSD and veterans without PTSD.

	Non-PTSD (N = 187)			PTSD (N = 250)				
	Range	Median	Mean (SD)	Range	Median	Mean (SD)	U Statistic	Probability
Erectile function	1–30	26	22.74 (8.33)	1–30	18	15.9 (9.71)	15,004	<0.001
Orgasmic function	1–10	9	8.09 (2.65)	1–10	6	5.7 (3.28)	15,200.5	<0.001
Sexual desire	2–10	8	7.72 (1.99)	2–10	6	5.7 (2.47)	14,832.5	<0.001
Intercourse satisfaction	0–15	10	9.2 (3.89)	0–15	7	6.4 (4.65)	17,465.5	<0.001
Overall satisfaction	2–10	8	7.83 (1.92)	2–10	6	6.3 (2.38)	16,139.5	<0.001
Premature ejaculation	0–17	4	5 (4.27)	0–20	7	7.2 (5.08)	18,229.5	<0.001

Erectile function n = 246; orgasmic function n = 240; sexual desire n = 244; intercourse satisfaction n = 242; overall satisfaction n = 236; and premature ejaculation n = 232.

**Table 2:** Descriptives and group comparison of medication consumption.

	All *n = 437*	PTSD NO *n = 187*	PTSD YES *n = 250*	Fisher’s Exact Test
At Least One Medication	N (%)	N (%)	N (%)	*p*-Value
Antidepressant Antipsychotic Hypnotic Anxiolytic Antiepileptic Antihypertensive Lipid-modifying agent Drugs used in diabetes H2 blockers Antihistamine	133 (30.4) 63 (14.4) 73 (16.7) 202 (46.2) 90 (20.6) 116 (26.5) 46 (10.5) 26 (5.9) 6 (1.4) 5 (1.1)	3 (1.6) 1 (0.5) 4 (2.1) 5 (2.7) 1 (0.5) 40 (21.4) 18 (9.6) 9 (4.6) 0 0	130 (52) 62 (24.8) 69 (27.6) 197 (78.8) 89 (35.6) 76 (30.4) 28 (11.2) 17 (6.8) 6 (2.4) 5 (2)	<0.001 <0.001 <0.001 <0.001 <0.001 0.038 0.639 0.421 0.04 0.07

**Table 3:** Correlation coefficients (overall sample).

	Erectile Function	Orgasmic Function	Sexual Desire	Intercourse Satisfaction	Overall Satisfaction	Premature Ejaculation
Age	−0.008	0.013	0.026	−0.033	0.044	−0.016
Educational level	0.07	0.05	0.057	0.02	0.022	−0.062
Socioeconomic level	0.187 **	0.167 **	0.102 *	0.145 **	0.133 **	0.017
In relationship, current ^1^	0.363 **	0.289 **	0.291 **	0.375 **	0.283 **	0.135 **
MDE, current	−0.236 **	−0.247 *	−0.206 **	−0.240 *	−0.172 **	0.084
MDE, lifetime	−0.193 **	−0.195 **	−0.189 **	−0.183 **	−0.122 *	0.083
Suicidality (low risk)	−0.114 *	−0.112 *	−0.144 *	−0.142 **	−0.085	0.058
Panic disorder, current	−0.107 *	−0.107 *	−0.140 **	−0.119 *	−0.102 *	0.093
Panic disorder, lifetime	−0.201 **	−0.199 **	−0.199 **	−0.159 **	−0.169 **	0.116 *
Other anxiety disorders	−0.164 **	−0.156 **	−0.152 **	−0.172 **	−0.181 **	0.092
Alcohol use disorders	−0.075	−0.040	−0.110 *	−0.073	−0.079	0.038
Diabetes mellitus	−0.050	−0.008	−0.008	−0.035	0.018	0.141 **
Hypertension (essential)	−0.210 **	−0.180 **	−0.149 **	−0.176 **	−0.176 **	0.141 **
Hyperplasia of prostate	−0.065	−0.046	−0.054	−0.112 *	−0.118*	0.018
Dis. of lipoprotein metabolism ^2^	−0.083	−0.075	−0.035	−0.046	0.001	0.035
Antidepressants	−0.310 **	−0.357 **	−0.316 **	−0.262 **	−0.237	0.144 **
Antipsychotics	−0.170 **	−0.179 **	−0.139 **	−0.118 *	−0.109 *	0.033
Hypnotics	−0.202 **	−0.199 **	−0.187 **	−0.178 **	−0.167 **	0.024
Anxiolytics	−0.319 **	−0.304 **	−0.323 **	−0.255 **	−0.284 **	0.152 **
Antiepileptics	−0.158 **	−0.155 **	−0.185 **	−0.138 **	−0.141 **	0.164 **
Antihypertensives	−0.118 *	−0.082	−0.096 *	−0.107 *	−0.133 **	0.005
Drugs used in diabetes	−0.060	−0.050	−0.021	−0.078	0.022	−0.008
Lipid-modifying agents	−0.100 *	−0.053	−0.060	−0.073	−0.110 *	0.006
Total PTSP symptoms	−0.434 **	−0.394 **	−0.437 **	−0.358 **	−0.401 **	0.255 **
War deployment *(in months)*	0.001	0.006	−0.023	0.054	0.019	0.159 **

^1^ Married and in cohabitation; ^2^ disorders of lipoprotein metabolism, MDE—major depressive episode. * *p* < 0.05; ** *p* < 0.01; Positive coefficients for erectile function, orgasmic function, sexual desire, intercourse satisfaction, overall satisfaction all imply better sexual functioning. Positive coefficients with premature ejaculation imply lower scores/more problems.

## Data Availability

The data presented in this study are available upon request from the corresponding author (M.L.-C.).
